# Valorization of Dried Okara Hydrolysate for Polyhydroxybutyrate Production by Newly Isolated *Burkholderia* sp. EP10

**DOI:** 10.3390/bioengineering13030313

**Published:** 2026-03-09

**Authors:** Eun Pyo Hwang, Do Young Kim, Jong-Sik Kim, Chung-Wook Chung

**Affiliations:** 1 Department of Biological Sciences, Gyeongkuk National University, Andong 36729, Republic of Korea; hwp1997@naver.com (E.P.H.); jsk@anu.ac.kr (J.-S.K.); 2 Microbiome Convergence Research Center, Korea Research Institute of Bioscience and Biotechnology (KRIBB), Daejeon 34141, Republic of Korea; kdy119@kribb.re.kr

**Keywords:** *Burkholderia* sp. EP10, lignocellulosic hydrolysate, okara byproduct, pH-uncontrolled fermentation, polyhydroxybutyrate

## Abstract

Dried okara (DOK), a lignocellulosic byproduct from tofu production, was evaluated as both a carbon source and culture medium to enable cost-effective polyhydroxybutyrate (PHB) production. Hydrolysis with either HCl or H_2_SO_4_ generated 48–51 g/L reducing sugars with peak values reaching 60.2 g/L using 3% acid at 121 °C. Analysis of monosaccharides indicated pentoses, especially xylose, as the main sugars present. A novel strain, *Burkholderia* sp. EP10 exhibited direct growth and PHB accumulation in DOK hydrolysate without requiring detoxification, tolerating inhibitory compounds such as furfural and 5-hydroxymethylfurfural. In shake flask experiments, the strain achieved 6.9 g/L biomass and 26.3 wt% PHB, while in fermentor studies, biomass reached 10.9 g/L and PHB content was 29.3 wt% at a C/N ratio of 5.7. Notably, these outcomes were achieved without pH control, constituting a key benefit for operational simplification and cost minimization. The biopolymer was verified as PHB using gas chromatography, Fourier transform infrared spectroscopy, and proton nuclear magnetic resonance spectroscopy. The PHB displayed melting transitions at 163.5 and 172.4 °C, a degradation onset at 268 °C, and high molecular weight (4.66 × 10^5^ Da). *Burkholderia* sp. EP10 for sustainable PHB production via direct bioconversion of lignocellulosic hydrolysates, without the need for pH adjustment, detoxification, or complex medium development.

## 1. Introduction

Petroleum-based plastics have been widely utilized across various industries due to their exceptional mechanical properties, including strength, flexibility, and thermal stability [1,2]. Nevertheless, the low rate of recycling for these materials has resulted in the significant accumulation of plastic waste, with an estimated 4.8–12.7 million tons entering the oceans every year. In the absence of more effective waste management strategies, the total amount of plastic waste is expected to exceed 1.3 gigatons by 2040 [3,4]. Ongoing dependence on fossil fuels for plastic manufacturing not only exhausts natural reserves but also imposes substantial environmental and ecological harm [5].

To tackle these pressing issues, considerable research efforts have been devoted to the advancement of sustainable and eco-friendly alternatives [6]. Among available bioplastics, which may be bio-based, biodegradable, or both, microbial polyhydroxyalkanoates (PHAs) have emerged as particularly promising. PHAs are intracellular polyesters produced by a range of microorganisms under nutrient-limited conditions, and these polymers are readily degraded by different microbes in terrestrial as well as marine environments [7,8]. The physicochemical properties of PHAs can be tailored by adjusting the monomer composition, making them strong candidates to substitute for petroleum-based plastics.

Polyhydroxybutyrate (PHB), the most extensively studied PHA, possesses thermal and crystalline characteristics similar to those of polypropylene, while also being fully biodegradable and biocompatible. These advantageous attributes render PHB suitable for diverse applications, including food packaging, agriculture, environmental remediation, and biomedicine [9,10]. Despite these advantages, broader industrial adoption of PHB is constrained by high production costs, with carbon substrates accounting for a substantial proportion of overall expenses [11]. Although fermentation strategies and strain optimization have enhanced PHB production performance under controlled conditions [12], large-scale implementation remains economically constrained, particularly due to substrate-related costs [13]. In addition to economic limitations, maintaining stable PHB accumulation when renewable or waste-derived substrates are employed presents further challenges. Variation in substrate composition and strain-specific metabolic responses can affect polymer yield and process reproducibility during scale-up [12]. For these reasons, increasing attention has been directed toward identifying stable and low-cost renewable feedstocks. Lignocellulosic biomass, particularly agricultural by-products, represents a promising renewable resource, as hydrolysis generates fermentable sugars that can be converted into PHB via microbial metabolic pathways [14]. Agave-derived materials, including syrups and lignocellulosic residues, have also been highlighted as sustainable substrates within circular economy frameworks [15].

Soybean (*Glycine max*), one of the world’s most important legumes, generates significant agro-residues during food processing. Okara, a by-product of tofu manufacturing, is characterized by a high moisture content that predisposes it to rapid spoilage, resulting in limited value-added use and frequent disposal [16]. When dried, okara (DOK) is converted into a stable biomass that contains cellulose, hemicellulose, proteins, lipids, and minerals, thus enhancing its suitability for various industrial applications [17,18]. Numerous studies have underscored this potential by demonstrating the utility of DOK in eco-friendly materials, including cellulose-based hydrogels [19] and films reinforced with dietary fiber [20]. Nevertheless, relatively few studies have examined the direct use of okara as a substrate for microbial PHB biosynthesis. In this research, we examined whether hydrolysates produced from DOK could function not only as a cost-effective carbon source but also as a culture medium for PHB biosynthesis using a newly isolated strain, EP10.

## 2. Materials and Methods

### 2.1. Preparation of Reducing Sugars

Wet okara, provided by Andong Pusansikpum (a handmade tofu producer using *Glycine max*), was dried at 100 °C for 24 h and ground into powder using a vacuum blender (CompLife, Incheon, Republic of Korea). Hydrolysis was performed with 130 g/L of dried okara (DOK) using various agents (NaOH, KOH, HCl, H_2_SO_4_) and concentrations (1–3% *v*/*v*), and reaction times (15–90 min). The standard hydrolysis condition was 13% (*w*/*v*) DOK treated with 2% H_2_SO_4_ at 121 °C for 90 min. After thermochemical hydrolysis, mixtures were neutralized with 3 M NaOH and centrifuged at 8000× *g* for 20 min at 4 °C. The resulting supernatant, referred to as DOK hydrolysate (DOKH), containing reducing sugars, was used as a carbon source or medium for strain isolation, flask culture, and aerobic fermentation.

### 2.2. Isolation of PHB Producing Bacterial Strain

Soil samples were collected from the Gilan-myeon area of Andong, Korea, to isolate novel PHB-producing bacteria. Samples were enriched in mineral salts medium (MSM) supplemented with reducing sugars derived from DOKH (RS_DOKH). Enrichment was carried out through three successive transfers, after which bacterial colonies were isolated. Selected strains were grown in MSM at 30 °C and stored in 50% glycerol at −80 °C. Specific details regarding media composition and enrichment procedures are described in the Supplementary Methods. Genomic DNA of the selected isolate was extracted after 24 h cultivation in Luria-Bertani (LB) medium using the DNeasy Plant Mini Kit (Qiagen, Hilden, Germany). The 16S rRNA gene was amplified using primers 518F (5′-CCAGCAGCCGCGGTAATACG-3′) and 800R (5′-TACCAGGGTATCTAATCC-3′) under standard polymerase chain reaction conditions: 94 °C for 30 s, 55 °C for 30 s, and 72 °C for 1 min for 28 cycles. Sequencing was performed by Macrogen Inc. (Seoul, Republic of Korea), and the sequence was compared with NCBI GenBank entries using BLAST (version 2.13.0) for identification. Alignment was conducted in MEGA X (version 10.2.6) using ClustalW, and a phylogenetic tree was constructed with 1000 bootstrap replications.

### 2.3. Medium Optimization for Cell Growth and PHB Production

To identify the optimal medium for PHB production, several formulations were evaluated: MSM, a modified MSM for *Pseudomonas putida* (MS-E*), a medium designed for *Ralstonia eutropha* H16 (MS-H16), yeast peptone meat medium (YPM), and DOKH used directly as a medium (DOKHM). All media were adjusted to pH 7.0 and supplemented with 20 g/L RS_DOKH. Inocula were prepared in LB medium, and 10% (*v*/*v*) of cultures at OD_660_ of 0.9–1.0 were transferred into each test medium. Cultivation took place at 30 °C and 200 rpm for 72 h. Comprehensive media composition details appear in the Supplementary Methods.

### 2.4. Flask Culture Condition

To optimize conditions for cell growth and PHB synthesis by strain EP10, cultivation was conducted in a 500-mL Erlenmeyer flask containing DOKHM supplemented with 20 g/L RS_DOKH (C/N ratio 40, *w*/*w*). Baseline incubation was performed at 30 °C, pH 7.0, and 200 rpm. The investigated variables included pH (3–11), temperature (10–40 °C), agitation (50–250 rpm), RS_DOKH concentration (10–30 g/L), culture duration (6–120 h), and C/N ratio (4–40, *w*/*w*).

### 2.5. Batch Fermentation of Strain Ep10

Based on the optimized flask parameters, a 3 L jar fermentor (Biofors, Buchen, Republic of Korea) with a 2 L working volume of DOKHM (20 g/L RS_DOKH) was operated at 25 °C, 200 rpm, and 1 vvm aeration. Three fermentation modes were investigated: (1) C/N ratio 40 (*w*/*w*) with pH maintained at 6.0; (2) C/N ratio 40 (*w*/*w*) without pH control; and (3) C/N ratio 5.7 (*w*/*w*) without pH control. For pH regulation, 1N NaOH and 1N HCl were added as required. Strain EP10 was pre-cultured in LB medium, and 10% (*v*/*v*) of cultures at OD_660_ 0.9–1.0 were used to inoculate the fermentor. At 6–12 h intervals, 20 mL samples were centrifuged at 17,000× *g* for 15 min. The supernatant was used for carbon and nitrogen quantification, while the pellet was dried at 60 °C for 48 h to assess biomass and PHB content. The PHB yield (%) was calculated as the ratio of PHB produced (g) to RS_DOKH consumed (g), multiplied by 100.

### 2.6. Isolation and Purification of PHB

PHB produced by strain EP10 was extracted from freeze-dried biomass utilizing hot chloroform in a Soxhlet apparatus. For purification, the crude PHB extract was gradually added to chilled methanol with vigorous stirring within a fume hood, resulting in precipitation. This purification sequence was repeated at least three times. The purified product was left in the fume hood for three days to fully evaporate residual organic solvents before subsequent characterization.

### 2.7. Analytical Methods

The monosaccharide composition of DOKH and the residual sugars present in the culture supernatant was determined using high performance liquid chromatography (HPLC) equipped with a refractive index detector. Quantification of potential inhibitors, including 5-hydroxymethylfurfural (5-HMF) and furfural, was also performed. Comprehensive analytical parameters are presented in the Supplementary Methods.

Total nitrogen content in DOKH or the culture supernatant was quantified by the Kjeldahl method using Nessler’s reagent (Kanto chemical, Tokyo, Japan). Sample aliquots (5 mL) were digested in concentrated H_2_SO_4_ with a K_2_SO_4_/CuSO_4_ (10:1, *w*/*w*) catalyst for 90 min, diluted to 50 mL, and distilled into a boric acid–sodium borate buffer (pH 9.5). The resulting distillate was reacted with Nessler’s reagent and its absorbance was measured at 490 nm. Calibration was performed using ammonium sulfate standards [21].

The elemental composition of DOKH was determined using inductively coupled plasma optical emission spectrometry (ICP-OES) (Agilent 5110, Agilent Technologies, Santa Clara, CA, USA). Additional details regarding the procedures can be found in the Supplementary Methods.

Intracellular PHB was quantified via acidic methanolysis of lyophilized cells, with methyl esters analyzed by gas chromatography (GC) equipped with a flame ionization detector. Monomer composition was further confirmed by gas chromatography–mass spectrometry (GC–MS). Fourier Transform Infrared (FT–IR) spectra of purified PHB were recorded using a PerkinElmer Paragon 1000 FT-IR spectrometer (PerkinElmer, Waltham, MA, USA), and structural attributes were evaluated by proton nuclear magnetic resonance (^1^H NMR) (600 MHz, Bruker AVANCE III) (Bruker Corp., Billerica, MA, USA). Commercial PHB (Sigma-Aldrich, St. Louis, MO, USA) served as the reference. Further experimental details are described in the Supplementary Methods.

Thermal transitions of the polymer were analyzed by differential scanning calorimetry (DSC) with a DSC 200 PC Phox instrument (Netzsch-Gerätebau GmbH, Selb, Germany), while thermal stability was evaluated by thermogravimetric/differential thermal analysis (TG/DTA) using a TG-DTA 8122 analyzer (Rigaku Corp., Tokyo, Japan). Weight-average molecular weight (*M_w_*), number-average molecular weight (*M_n_*), and polydispersity index (PDI) of PHA were determined by size exclusion chromatography (SEC) on a Waters Alliance e2695 system (Waters Corporation, Milford, MA, USA) equipped with a refractive index detector, with calibration based on polystyrene standards. Detailed experimental parameters are listed in the Supplementary Methods.

## 3. Results and Discussion

**Figure 1:**
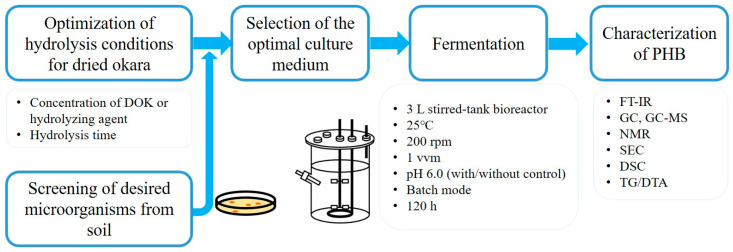
Schematic overview of the experimental design for PHB production from DOK, including hydrolysis optimization, culture medium selection, fermentation in a 3 L stirred-tank bioreactor (25 °C, 200 rpm, 1 vvm, pH 6.0, 120 h), and physicochemical characterization.

The overall experimental design of this study is summarized in Figure 1.

### 3.1. Optimization of Hydrolysis Conditions for DOK

**Figure 2:**
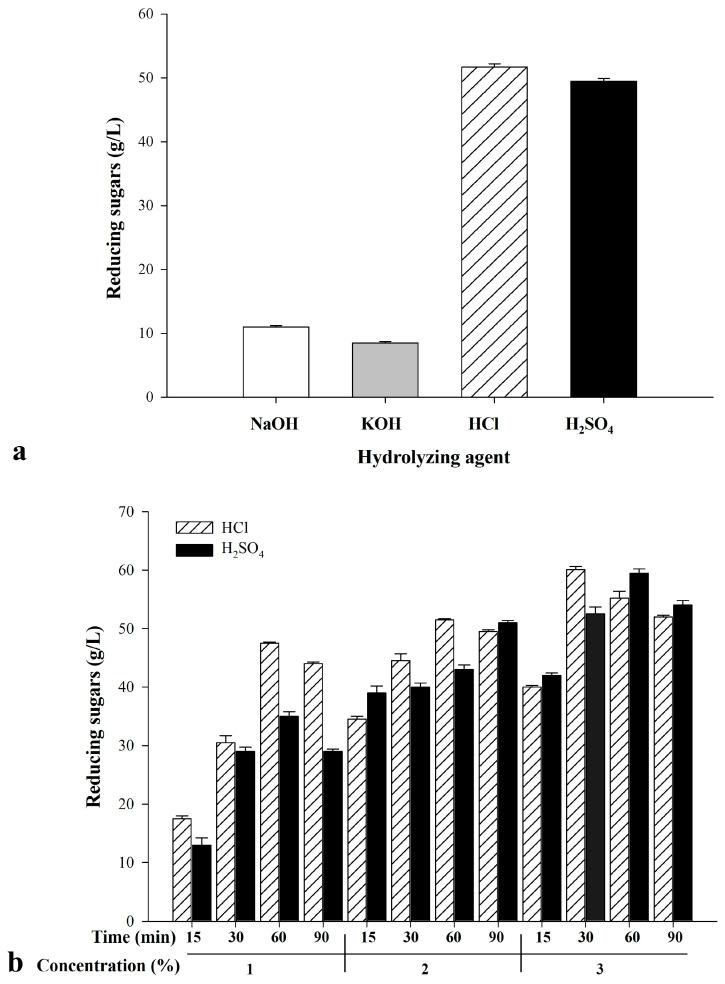
Comparison of reducing sugars concentrations obtained under distinct hydrolysis parameters: (**a**) effect of acid type, (**b**) effects of hydrolysis duration and acid concentration. The initial DOK concentration was maintained at 130 g/L.

To establish optimal hydrolysis conditions for DOK, we first performed a baseline treatment using 13% (*w*/*v*) DOK suspended in 2% (*v*/*v*) hydrolyzing agent at 121 °C for 90 min. Subsequently, various process parameters were systematically investigated to maximize sugar yield. Acidic agents (HCl, H_2_SO_4_) produced 48–51 g/L reducing sugars, which was almost five times higher compared to alkaline agents (NaOH, KOH) (Figure 2a). Importantly, these yields were obtained without additional pretreatment. The higher saccharification efficiency with acid likely arises from their ability to depolymerize hemicellulose into monomeric sugars directly, whereas alkaline conditions may further convert liberated sugars into short-chain acids and aldehydes, reducing the measured reducing-sugar yield [22,23,24]. Optimization efforts then focused on acid hydrolysis. Increasing acid concentration and reaction time improved sugar release, though the specific acid used had minimal impact (Figure 2b). When the hydrolysis duration exceeded 60 min, sugar concentration decreased, most likely due to the degradation of acid-labile sugars [25,26]. The highest yields (60.2 and 59.5 g/L) were observed with 13% (*w*/*v*) DOK treated with 3% HCl or H_2_SO_4_ at 121 °C for either 30 or 60 min.

The monosaccharide composition of DOKH is summarized in Table 1. When treated with 3% (*v*/*v*) HCl, arabinose constituted approximately 42% of the total reducing sugars, with pentoses making up 57.7%. Likewise, hydrolysis using 3% (*v*/*v*) H_2_SO_4_ resulted in about 50% xylose, and pentoses accounted for 66% of the total reducing sugars. This strong prevalence of pentoses suggests preferential breakdown of hemicellulose, which is more acid-sensitive than crystalline cellulose [25,26]. Dilute H_2_SO_4_ hydrolysis was found to be more effective than HCl in generating xylose. Several previous studies have demonstrated that H_2_SO_4_ treatment produces higher yields of monomeric sugars than HCl, suggesting that H_2_SO_4_ acts as a more efficient catalyst for hemicellulose hydrolysis. However, H_2_SO_4_ hydrolysis also generates higher concentrations of inhibitors such as furfural and 5-HMF, likely due to accelerated sugar degradation under strong acidic conditions [27,28].

### 3.2. Isolation and Identification of PHB-Producing Bacterial Strain

**Figure 3:**
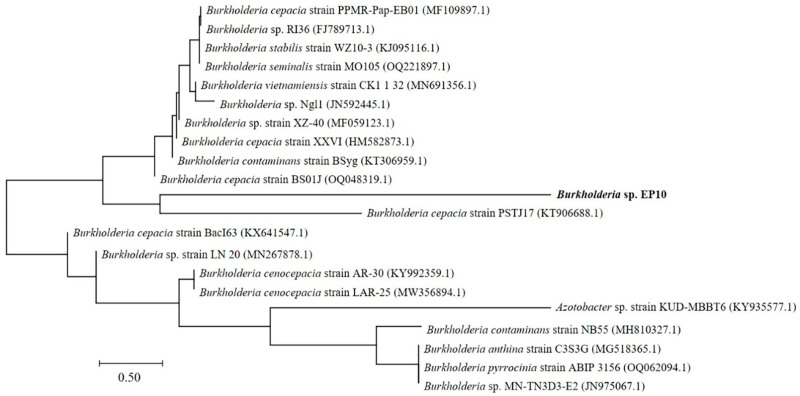
Phylogenetic analysis based on the 16S rDNA sequence of strain EP10. The tree was generated using the neighbor-joining method in MEGA X following ClustalW multiple sequence alignment, with bootstrap values calculated from 1000 replicates.

To isolate PHB-producing bacteria, RS_DOKH was obtained by hydrolyzing 13% DOK with 3% (*v*/*v*) H_2_SO_4_ for 60 min at 121 °C. Enrichment subculturing was subsequently performed three times by inoculating MSM containing 20 g/L RS_DOKH with 10 g soil sample. A total of 40 PHA-producing bacterial strains were isolated from the culture, and strain EP10 was chosen based on its superior cell proliferation and PHB production. Identification by 16S rDNA sequencing and BLAST analysis revealed 98.57% similarity to *Burkholderia cepacia* PSTJ17, placing the isolate within the *Burkholderia* clade (Figure 3). The strain was assigned the designation *Burkholderia* sp. EP10.

*Burkholderia* sp. EP10 was cultivated in MSM supplemented with 20 g/L RS_DOKH at pH 7.0 and 30 °C for 72 h, utilizing hydrolysates derived from either HCl or H_2_SO_4_ as the carbon source. As shown in Table 2, despite the higher levels of inhibitory compounds (especially furfural) present in the xylose-rich H_2_SO_4_ hydrolysate, both cell growth and PHB accumulation were greater than those measured in the arabinose-rich HCl medium. In particular, *Burkholderia* sp. EP10 achieved approximately 50% higher biomass with the H_2_SO_4_-derived hydrolysate, while PHB accumulation remained comparable between treatments. This observation may be attributed to the strain’s adaptation to xylose-rich substrates, given that its original isolation source was xylose-rich hydrolysate. Accordingly, xylose-rich RS_DOKH was selected for use in subsequent experiments. The C/N ratio of this hydrolysate was 40, and its elemental composition is presented in Table S1. Collectively, these results indicate that xylose is a preferable carbon source for *Burkholderia* sp. EP10 to support growth and PHB biosynthesis, in agreement with studies showing that pentose-utilizing *Burkholderia* strains yield increased PHA under xylose-rich conditions [29].

### 3.3. Screening and Optimization of Culture Media for Cell Growth and PHB Accumulation

**Figure 4:**
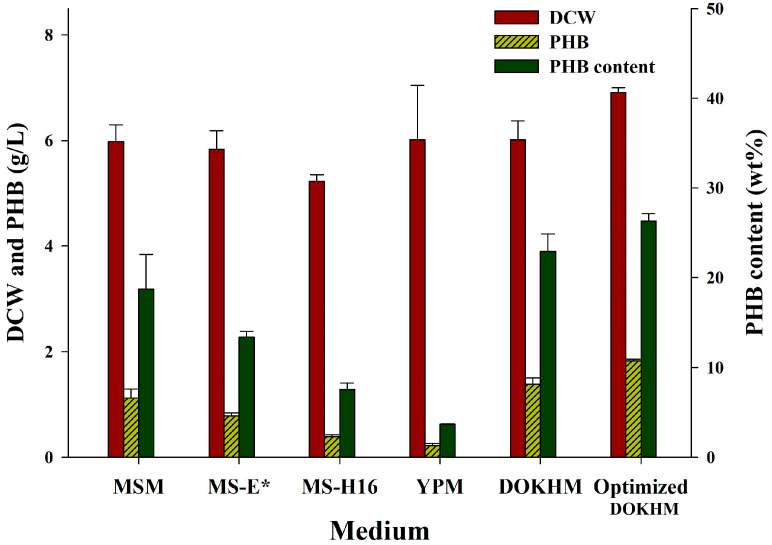
Comparison of cell growth and PHB production by *Burkholderia* sp. EP10 across different media, as well as before and after process optimization. Cultivation was conducted at pH 7, 30 °C, 200 rpm for 72 h with RS_DOKH at 20 g/L. MS-E* denotes the name of the medium used in this study.

To determine the optimal growth medium for *Burkholderia* sp. EP10, three defined media, YPM, and DOKH—(the latter serving both as the carbon source and as the medium, referred to as DOKHM)—were assessed. The standard condition employed a pH of 7.0, temperature of 30 °C, agitation at 200 rpm, and 20 g/L RS_DOKH with a C/N ratio of 40. As depicted in Figure 4, cell biomass, expressed as dry cell weight (DCW g/L), remained consistent across MSM, MS-E*, YPM, and DOKHM (with the exception of MS-H16), while DOKHM resulted in the greatest PHB accumulation. Under cultivation with DOKHM, *Burkholderia* sp. EP10 attained 6.01 g/L biomass and 22.92 wt% PHB. Consequently, DOKHM containing 20 g/L reducing sugars was chosen as the standard culture medium for further investigations.

Optimization of culture conditions was further conducted in DOKHM. Parameters assessed included pH (3.0–11.0), temperature (10–40 °C), agitation speed (50–250 rpm), reducing sugars concentrations (5–30 g/L), and C/N ratio (4–40) (Figure S1). Additionally, MSM supplemented with xylose (20 g/L) was employed to evaluate the impact of inhibitory compounds (furfural, 5-HMF) at various concentrations. Among all tested conditions, the combination of pH 6.0, 25 °C, 200 rpm, 20 g/L reducing sugars, and a C/N ratio of 5.7 yielded the most favorable results for both cell growth (6.91 g/L) and PHB accumulation (26.3 wt%) (Figure 4).

A significant outcome of this study is that *Burkholderia* sp. EP10 was capable of both growth and PHB accumulation even when untreated hydrolysates were used. In DOKHM containing 20 g/L reducing sugars, the levels of furfural and 5-HMF present did not notably affect cell growth (Figure S1). This is in accordance with earlier findings that *Burkholderia cepacia* exhibited considerable resistance to furfural, with a minimum inhibitory concentration reported at 6.0 g/L [30]. Therefore, it can be deduced that at lower concentrations, such as approximately 1.0 g/L, the bacterium’s growth and PHB accumulation are not substantially hindered. Given that the inhibitor concentrations observed in this study were below this critical value, the continued growth of *Burkholderia* sp. EP10 can be well accounted for. Taken together, *Burkholderia* sp. EP10 displays notable metabolic adaptability under inhibitory conditions, highlighting its promise for industrial applications. This inherent tolerance may reduce the requirement for detoxification procedures, potentially lowering production costs and streamlining processes in sustainable PHB manufacturing.

### 3.4. Fermentation Under Three Culture Conditions

**Figure 5:**
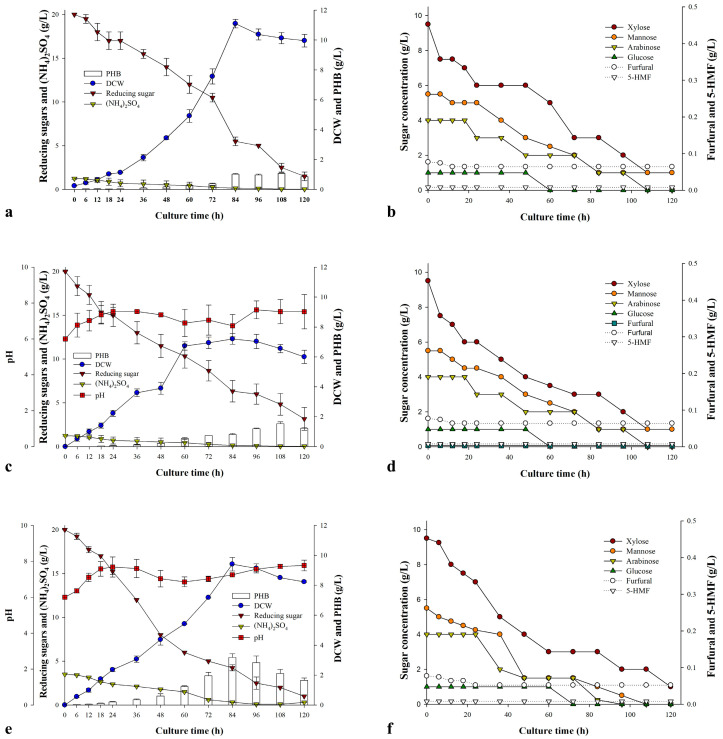
PHB biosynthesis by *Burkholderia* sp. EP10 cultivated in DOKHM under various pH 6 regulation strategies. Panels (**a**,**c**,**e**) illustrate cell growth and PHB accumulation under three fermentation conditions: (**a**) pH-controlled (pH 6, 120 h, 200 rpm, 25 °C, 20 g/L RS_DOKH, C/N ratio 20, 1 vvm), (**c**,**e**) non-controlled fermentations initiated at pH 6 with no further adjustment, and distinct C/N ratios (20 and 5.7). Panels (**b**,**d**,**f**) display the respective consumption profiles of sugars and growth inhibitors.

Due to the differing cultivation conditions between the flask and fermentor, the optimized culture parameters—including carbon concentration, temperature, and pH—were adjusted accordingly, except for the C/N ratio. In fermentor cultures using DOKHM (20 g/L reducing sugars, pH 6.0, 25 °C) without additional nitrogen supplementation, *Burkholderia* sp. EP10 achieved a maximum biomass of 9.4 g/L at 84 h. At 108 h, the PHB content reached 13.2 wt%, corresponding to a PHB yield of 7.5% based on the amount of RS_DOKH consumed (Figure 5a). Importantly, PHB accumulation was 50% lower than that observed in flask culture. A lag phase was observed until 24 h, after which substantial PHB accumulation was detected beginning at 72 h. Xylose and mannose were preferentially consumed, whereas arabinose and glucose utilization occurred after 18 h and 48 h, respectively (Figure 5b).

The second fermentation was conducted with the medium initially set at pH 6.0, which subsequently remained uncontrolled during cultivation and stabilized near-neutral (pH 7.0–8.0). Under these conditions, the biomass reached 8.2 g/L, which was lower than that in pH-controlled fermentation; however, the PHB content increased to 19.7 wt% at 108 h, corresponding to a PHB yield of 9.25% (Figure 5c). The shortened lag phase facilitated earlier xylose consumption, whereas the overall sugar utilization patterns remained largely consistent regardless of pH control (Figure 5d).

When ammonium sulfate was added to reduce the C/N ratio to 5.7, *Burkholderia* sp. EP10 exhibited optimal production. Biomass (10.9 g/L) and PHB content (29.3 wt%) were reached at 84 h, corresponding to a PHB yield of 19.5%, whereas PHB accumulation was initiated as early as 18 h (Figure 5e). For all tested conditions, xylose and mannose were consumed prior to significant arabinose and glucose uptake (Figure 5b,d,f).

The fermentor experiments highlighted specific tradeoffs among the three cultivation strategies.

Under pH-controlled conditions, biomass production was enhanced but increased PHB accumulation was restricted. Conversely, cultures operated without pH control exhibited reduced biomass but increased PHB accumulation, which we propose may be associated with accelerated xylose assimilation. The influence of pH regulation is system- and strain-dependent: for *Bacillus megaterium*, maintaining neutral pH improved both biomass and PHB accumulation compared to uncontrolled flasks [31], whereas mixed microbial cultures produced inconsistent outcomes. Ref. [32] reported higher PHA content without pH regulation, while ref. [33] did not observe notable differences. Notably, in this study, the medium’s pH remained approximately neutral, implying that *Burkholderia* sp. EP10 might depend on alternative metabolic pathways instead of relying on acidification.

The nitrogen-rich condition (C/N 5.7) yielded the highest performance, demonstrating that an increased nitrogen supply not only promoted cell growth but also stimulated PHB accumulation. This result is in contrast to the traditional view that nitrogen limitation enhances PHB production and suggests that *Burkholderia* sp. EP10 utilizes a unique regulatory pathway.

Analysis of sugar consumption established *Burkholderia* sp. EP10’s preference for xylose and mannose, consistent with trends reported for other PHA-producing bacteria. For instance, *Priestia* sp. strain JY310 and *Schlegelella thermodepolymerans* DSM 15344 displayed similar responses to pH [21,34], while *Paracoccus* sp. LL1 and *Paraburkholderia sacchari* IPT 101 showed stepwise sugar utilization from complex hydrolysates, first consuming glucose and subsequently metabolizing other sugars [35,36]. Remarkably, *S. thermodepolymerans* DSM 15344 produced up to 61% PHAs of DCW on undetoxified xylose-rich hydrolysates, indicating that rapid xylose utilization and metabolic efficiency are critical for maximizing PHA yields. *Burkholderia* sp. EP10 demonstrates a similar phenotype, favoring xylose and supporting robust PHB accumulation (Table 3).

Overall, *Burkholderia* sp. EP10 achieved 6.9 g/L biomass in flask cultures, which increased to 10.9 g/L under fermentor conditions, accompanied by improved PHB production. The PHB concentration reached 3.18 g/L under fermenter conditions, demonstrating scalable production performance comparable to previously reported hydrolysate-based systems. Although the intracellular PHB content (29.3 wt%) was lower than that of high-PHB-accumulating strains such as *P. sacchari* IPT 101, it remained within the range reported for various waste-derived substrates. Importantly, *Burkholderia* sp. EP10 was capable of directly utilizing DOKH medium without detoxification or nutrient supplementation, highlighting its robustness and potential for cost-effective PHB production from heterogeneous agricultural byproducts. These findings support the feasibility of further process optimization for sustainable biopolymer production from low-cost waste streams.

### 3.5. Structural and Physicochemical Characterization of PHB

**Figure 6:**
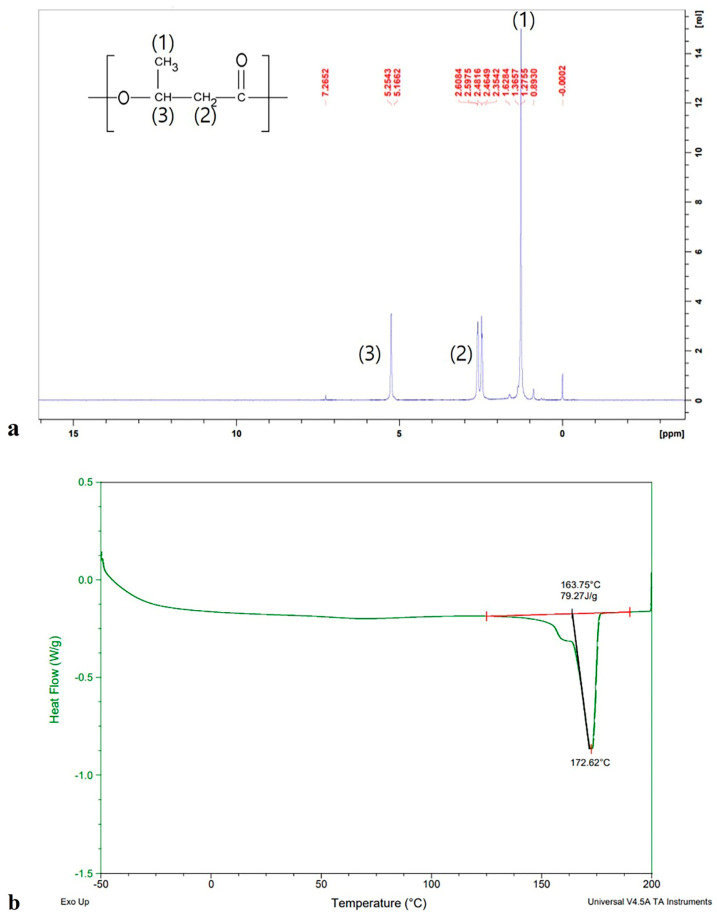
Structural and thermal analysis of PHB synthesized by *Burkholderia* sp. EP10. (**a**) ^1^H NMR spectrum confirming polymeric structure. (**b**) DSC thermogram illustrating the thermal transition profile.

The polymer produced by *Burkholderia* sp. EP10 was confirmed as PHB using several analytical approaches. GC analysis exhibited a pronounced 3-hydroxybutyrate (3HB) signal at 5.07 min, while the benzoic acid internal standard appeared at 7.96 min (Figure S2a). Results from GC/MS further verified that the principal peak of the polymer matched the reference spectrum of PHB (Figure S2b). FT-IR spectra (4000–400 cm^−1^) showed characteristic absorption signals at 3436 cm^−1^ (–OH stretching), 2850–2975 cm^−1^ (C–H stretching), and approximately 1720 cm^−1^ (C=O stretching, ester functionality), all consistent with PHB structural features (Figure S2c). Additionally, ^1^H–NMR spectra contained the expected 3HB resonances at 1.275 ppm (–CH_3_), 2.464 ppm (–CH_2_), and 5.162 ppm (–CH), corroborating the polymer’s chemical identity (Figure 6a) [42].

A summary of thermal and molecular properties appears in Table 4. DSC analysis displayed two distinct melting endotherms at 163.5 °C and 172.4 °C with an undetectable glass transition temperature (*T_g_*), which is commonly observed in semicrystalline PHB (Figure 6b). TGA demonstrated an initial minor weight reduction (2.6%) up to 189.3 °C, followed by pronounced decomposition beginning at 267.5 °C, resulting in nearly 90% mass loss by 288.4 °C (Figure S3). SEC measurements determined a *M_w_* of 4.66 × 10^5^ Da, *M_n_* of 1.92 × 10^5^ Da, and a PDI of 2.42, indicating the polymer possessed a high-molecular-weight and a relatively uniform distribution (Figure S4).

The combined results from GC, FT-IR, and ^1^H–NMR analyses established that the polymer produced by *Burkholderia* sp. EP10 is PHB, exhibiting spectral characteristics identical to those of standard PHB. The twin melting peaks seen in the DSC analysis are characteristic of semicrystalline polymers and are attributed to lamellae of varying thickness as well as to the rearrangement of imperfect crystals during heating [45,46]. The slightly reduced *T_m_* in comparison to commercial PHB implies improved processability during thermal molding, which may present a benefit for industrial applications. TGA assessment revealed a higher degradation onset temperature than previously documented for PHBs, suggesting enhanced thermal stability and the potential for increased durability at elevated temperatures. Such enhanced stability broadens the range of potential uses where thermal resistance is essential. The polymer possessed a high Mw, a factor that is associated with increased toughness, improved impact resistance, and potential for application in medical or high-value fields [47].

The relatively narrow PDI in comparison to commercial PHB reflects a more uniform molecular weight distribution, which, based on polymer science principles, can result in more predictable thermal and mechanical properties [48]. This uniform distribution is especially important in biomedical contexts, where consistent material performance and reliable degradation rates are critical [47,49]. Thus, it can be inferred that the narrower PDI reported in this study would provide increased consistency in high-value or medical applications.

## 4. Conclusions

In this study, DOK, a lignocellulosic biomass, underwent acid hydrolysis for low-cost PHB production. The hydrolysate generated from 13% (*w*/*v*) DOK with 3% (*v*/*v*) H_2_SO_4_ effectively supported both substantial cell growth and PHB synthesis when utilized directly as the culture medium for the newly isolated *Burkholderia* sp. EP10. Without pH regulation and under a low C/N ratio, this strain efficiently consumed xylose and mannose from the earliest cultivation stages, producing PHB with superior thermal stability. Collectively, these findings highlight *Burkholderia* sp. EP10 as a strong candidate for PHB production in non-pH-controlled and nitrogen-rich environments. Fermenter cultivation achieved 10.9 g/L biomass and 3.18 g/L PHB, demonstrating scalable performance. The strain achieved PHB biosynthesis directly from lignocellulosic biomass hydrolysate with no pretreatment required. Particularly, these outcomes underscore promising prospects for industrial-scale PHA manufacturing using lignocellulosic byproducts.

E-supplementary data of this word can be found in the online version of the paper.

## Figures and Tables

**Table 1:** Sugar and growth inhibitor composition of DOKH under optimal hydrolysis conditions.

Parameter	HCl	H_2_SO_4_
	Concentration (g/L)	
Total sugars	60.17	59.53
Arabinose	25.17	9.70
Xylose	9.56	29.39
Mannose	18.72	16.82
Glucose	6.72	3.62
Growth inhibitors	0.26	1.70
Furfural	0.15	1.54
5-HMF	0.11	0.16

HMF, hydroxymethylfurfural.

**Table 2:** Cell growth and PHB accumulation of *Burkholderia* sp. EP10 utilizing RS_DOKH with various acid hydrolyzing agents.

Strain	Hydrolytic Agent	* DCW (g/L)	PHB Content (g/L)	PHB Yield (wt%)
*Burkholderia* sp. EP10	H_2_SO_4_	6.05	1.13	18.68
	HCl	4.07	0.74	18.20

* DCW, dry cell weight.

**Table 3:** Comparative analysis of PHB production by *Burkholderia* sp. EP10 and other bacterial strains utilizing lignocellulosic hydrolysates.

Strain	Carbon Source	DCW (g/L)	PHB (g/L)	PHB (wt%)	Culture Scale	Reference
*Burkholderia* sp. EP10	DOK hydrolysate medium	6.91	1.82	26.3	Flask	This study
		10.87	3.18	29.3	Fermenter	
*Priestia* sp. strain JY310	Rice husk hydrolysate	7.8	3.1	50.4	Batch fermenter	Lee, J. Y., et al., 2023 [21]
*Bacillus aerophilus* RSL-7	Bael fruit hydrolysate medium	12.5	1.275	10.1	fermenter	Sabapathy, P. C., et al., 2019 [37]
*Burkholderia cepacia* IPT 048	Sugarcane bagasse hydrolysate	4.4	2.3	53.0	Batch fermenter	Silva, L. F., et al., 2004 [29]
*Paraburkholderia sacchari* IPT 101	Softwood hemicellulose hydrolysates	7.1	5.7	80.5	Flask	Dietrich, K., et al., 2018 [38]
*Halomonas cupida* J9	Corn straw hydrolysate	7.0	2.45	35.0	Batch fermenter	Wang, S., et al., 2024 [39]
*Loktanella* sp. SM43	Pine tree hydrolysate	4.68	3.6	78.0	Flask	Lee, S. M., et al., 2022 [40]
*Halomonas halophila* CCM 3662	Spent coffee grounds hydrolysate	3.5	2.1	61.9	Flask	Kucera, D., et al., 2018 [41]

**Table 4:** Comparison of molecular weight and thermal properties of PHB synthesized by *Burkholderia* sp. EP10 and other bacterial strains from lignocellulosic hydrolysates.

Microbial Strain	Carbon Source	*T_g_* (°C)	*T_m_* (°C)	*T_d_* (°C)	*M_w_* (Da)	*M_n_* (Da)	*PDI* (*M_w_/M_n_*)	Reference
*Burkholderia* sp. EP10	DOKHM	N.I	172.4	288.4	465,918	191,930	2.42	This study
*Priestia* sp. strain JY310	Rice husk hydrolysate	N.I	167.9	268.1	76,800	16,239	4.73	Lee, J. Y., et al., 2023 [21]
*Sphingobium scionense* WP01	soft wood hydrolysate	1.7	176.7	N.I	1,138,000	186,000	6.1	Bowers et al., 2013 [43]
*Cupriavidus necator*	Rice husk hydrolysate	N.I	175.1	240.0	135,000	124,000	1.09	Zhang, Y., et al., 2020 [23]
*Priestia* sp. YH4	Sugar cane molasses	N.I	167.2	N.I	285,000	105,000	2.7	Jung et al., 2023 [44]
Standard PHB (Sigma-Aldrich)	N.I	N.I	169.0	N.I	429,000	319,000	1.34	Jung et al., 2023 [44]

## Data Availability

The original contributions presented in this study are included in the article/supplementary material. Further inquiries can be directed to the corresponding author.

## Supplementary Materials

The following supporting information can be downloaded at: https://www.mdpi.com/article/10.3390/bioengineering13030313/s1. Table S1: Elemental composition of DOKH obtained under optimal hydrolysis conditions (3% H_2_SO_4_, 121 °C, 60 min). Values represent the reducing sugar fraction measured following hydrolysis. Figure S1: Effects of culture conditions and lignocellulosic inhibitors on growth and PHB biosynthesis of *Burkholderia* sp. EP10. Cultivations were conducted in DOKHM under different (a) pH values, (b) temperatures, (c) agitation speeds, (d) reducing sugar concentrations, and (e) C/N ratios. For inhibitor tolerance analysis, cells were cultivated in MSM containing xylose (20 g/L) for 72 h with (f) furfural or (g) 5-HMF. Data represent cell growth, PHB content, and PHB concentration. Figure S2: Structural characterization of PHB isolated from *Burkholderia* sp. EP10 grown in DOKHM medium with a C/N ratio of 5.7, initiated at pH 6 without subsequent pH adjustment. Following fermentation and cell collection, PHB was extracted and subjected to (a) GC and (b) GC–MS analysis, employing benzoic acid as the internal standard for determining monomer composition, and (c) FT-IR spectroscopy to verify functional groups and structural features. Figure S3: Thermal stability assessment of PHB isolated from *Burkholderia* sp. EP10 grown in DOKHM medium at a C/N ratio of 5.7, initiated at pH 6 without further pH regulation. After fermentation and PHB isolation, TG–DTA analysis was conducted to assess the thermal degradation profile. Figure S4: Molecular weight profile of PHB isolated from *Burkholderia* sp. EP10 grown in DOKHM medium at a C/N ratio of 5.7, initiated at pH 6 without additional pH regulation. PHB samples were examined by SEC to assess *M_w_*, *M_n_*, and PDI.

## References

[1] Pawde S.V., Kaewprachu P., Kingwascharapong P., Sai-Ut S., Karbowiak T., Jung Y.H., Rawdkuen S. (2025). A comprehensive review on plant protein-based food packaging: Beyond petroleum-based polymers. Curr. Res. Food Sci..

[2] Hu S., Han L., Yu C., Pan L., Tu K. (2025). A Review on Replacing Food Packaging Plastics with Nature-Inspired Bio-Based Materials. Foods.

[3] Jambeck J.R., Geyer R., Wilcox C., Siegler T.R., Perryman M., Andrady A., Narayan R., Law K.L. (2015). Plastic waste inputs from land into the ocean. Science.

[4] Lau W.W.Y., Shiran Y., Bailey R.M., Cook E., Stuchtey M.R., Koskella J., Velis C.A., Godfrey L., Boucher J., Murphy M.B. (2020). Evaluating scenarios toward zero plastic pollution. Science.

[5] Chacón M., Wongsirichot P., Winterburn J., Dixon N. (2024). Genetic and process engineering for polyhydroxyalkanoate production from pre- and post-consumer food waste. Curr. Opin. Biotechnol..

[6] Huo G., Zhu Y., Liu Q., Tao R., Diao N., Wang Z., Chen T. (2017). Metabolic engineering of an *E. coli* ndh knockout strain for PHB production from mixed glucose–xylose feedstock. J. Chem. Technol. Biotechnol..

[7] Di Bartolo A., Infurna G., Dintcheva N.T. (2021). A review of bioplastics and their adoption in the circular economy. Polymers.

[8] Javaid H., Nawaz A., Riaz N., Mukhtar H., Ul-Haq I., Shah K.A., Khan H., Naqvi S.M., Shakoor S., Rasool A. (2020). Biosynthesis of polyhydroxyalkanoates (PHAs) by the valorization of biomass and synthetic waste. Molecules.

[9] Sirohi R., Pandey J.P., Gaur V.K., Gnansounou E., Sindhu R. (2020). Critical overview of biomass feedstocks as sustainable substrates for the production of polyhydroxybutyrate (PHB). Bioresour. Technol..

[10] Turco R., Santagata G., Corrado I., Pezzella C., Di Serio M. (2021). In vivo and Post-synthesis Strategies to Enhance the Properties of PHB-Based Materials: A Review. Front. Bioeng. Biotechnol..

[11] Zhang L., Jiang Z., Tsui T.H., Loh K.C., Dai Y., Tong Y.W. (2022). A Review on enhancing *Cupriavidus necator* fermentation for poly(3-hydroxybutyrate) (PHB) production from low-cost carbon sources. Front. Bioeng. Biotechnol..

[12] Ray S., Jin J.-O., Choi I., Kim M. (2023). Recent trends of biotechnological production of polyhydroxyalkanoates from C1 carbon sources. Front. Bioeng. Biotechnol..

[13] Koller M. (2018). Biodegradable and Biocompatible Polyhydroxyalkanoates (PHA): Auspicious microbial macromolecules for pharmaceutical and therapeutic applications. Molecules.

[14] Jaffur N., Jeetah P., Kumar G. (2021). A review on enzymes and pathways for manufacturing polyhydroxybutyrate from lignocellulosic materials. 3 Biotech.

[15] Martínez-Herrera R.E., Rutiaga-Quiñones O.M., Alemán-Huerta M.E. (2021). Integration of Agave plants into the polyhydroxybutyrate (PHB) production: A gift of the ancient Aztecs to the current bioworld. Ind. Crops Prod..

[16] Asghar A., Afzaal M., Saeed F., Ahmed A., Ateeq H., Shah Y.A., Islam F., Hussain M., Akram N., Shah M.A. (2023). Valorization and food applications of okara (soybean residue): A concurrent review. Food Sci. Nutr..

[17] O’Toole D.K. (1999). Characteristics and use of okara, the soybean residue from soy milk production-a review. J. Agric. Food Chem..

[18] Ashaolu T.J., Zhao G. (2020). Fabricating a pickering stabilizer from okara dietary fibre particulates by conjugating with soy protein isolate via maillard reaction. Foods.

[19] Cui X., Lee J.J.L., Chen W.N. (2019). Eco-friendly and biodegradable cellulose hydrogels produced from low cost okara: Towards non-toxic flexible electronics. Sci. Rep..

[20] Scarcella J.V., Lopes M.S., Silva E.K., Andrade G.S.S. (2024). Valorization of okara by-product for obtaining soluble dietary fibers and their use in biodegradable carboxymethyl cellulose-based film. Int. J. Biol. Macromol..

[21] Lee J.Y., Kim M.H., Kim J.S., Yun B.R., Kim D.Y., Chung C.W. (2023). Biotransformation of D-xylose-rich rice husk hydrolysate by a rice paddy soil bacterium, *Priestia* sp. Strain JY310, to low molecular weight poly (3-hydroxybutyrate). Biomolecules.

[22] Mosier N., Wyman C., Dale B., Elander R., Lee Y.Y., Holtzapple M., Ladisch M. (2005). Features of promising technologies for pretreatment of lignocellulosic biomass. Bioresour. Technol..

[23] Zhang Y., Wang L., Li T., Shen Y., Luo J. (2020). Acid soaking followed by steam flash-explosion pretreatment to enhance saccharification of rice husk for poly (3-hydroxybutyrate) production. Int. J. Biol. Macromol..

[24] Li Q., Jiang Y., Ren C., Jiang Q., Feng J., Wang M., Gao Z., Cao W. (2023). Effects of different hydrolysis methods on the hydrolysate characteristics and photo-fermentative hydrogen production performance of corn and sorghum straw. Energies.

[25] Carvalho D.M., Colodette J.L. (2017). Comparative study of acid hydrolysis of lignin and polysaccharides in biomasses. BioResources.

[26] Lin R., Deng C., Rajendran K., Bose A., Kang X., Murphy J.D. (2020). Competing reactions limit production of sugars in hydrothermal hydrolysis. Front. Energy Res..

[27] Lavarack B.P., Griffin G.J., Rodman D. (2002). The acid hydrolysis of sugarcane bagasse hemicellulose to produce xylose, arabinose, glucose and other products. Biomass Bioenergy.

[28] Oriez V., Peydecastaing J., Pontalier P.Y. (2019). Lignocellulosic biomass fractionation by mineral acids and resulting extract purification processes: Conditions, yields, and purities. Molecules.

[29] Silva L.F., Taciro M.K., Michelin Ramos M.E., Carter J.M., Pradella J.G.C., Gomez J.G.C. (2004). Poly-3-hydroxybutyrate (P3HB) production by bacteria from xylose, glucose and sugarcane bagasse hydrolysate. J. Ind. Microbiol. Biotechnol..

[30] Dietrich D., Illman B., Crooks C. (2013). Differential sensitivity of polyhydroxyalkanoate producing bacteria to fermentation inhibitors and comparison of polyhydroxybutyrate production from *Burkholderia cepacia* and *Pseudomonas pseudoflava*. BMC Res. Notes.

[31] Alkotaini B., Koo H., Kim B.S. (2016). Production of polyhydroxyalkanoates by batch and fed-batch cultivations of *Bacillus megaterium* from acid-treated red algae. Korean J. Chem. Eng..

[32] Montiel-Jarillo G., Carrera J., Suárez-Ojeda M.E. (2017). Enrichment of a mixed microbial culture for polyhydroxyalkanoates production: Effect of pH and N and P concentrations. Sci. Total Environ..

[33] Correa-Galeote D., Argiz L., Val del Rio A., Mosquera-Corral A., Juarez-Jimenez B., Gonzalez-Lopez J., Rodelas B. (2022). Dynamics of PHA-accumulating bacterial communities fed with lipid-rich liquid effluents from fish-canning industries. Polymers.

[34] Kourilova X., Pernicova I., Sedlar K., Musilova J., Sedlacek P., Kalina M., Koller M., Obruca S. (2020). Production of polyhydroxyalkanoates (PHA) by thermophilic *Schlegelella thermodepolymerans* DSM 15344 from xylose-rich substrates. Bioresour. Technol..

[35] Sawant S.S., Salunke B.K., Kim B.S. (2015). Degradation of corn stover by fungal cellulase cocktail for production of polyhydroxyalkanoates by moderate halophile *Paracoccus* sp. LL1. Bioresour. Technol..

[36] Dietrich K., Dumont M.J., Orsat V., Del Rio L.F. (2019). Consumption of sugars and inhibitors of softwood hemicellulose hydrolysates as carbon sources for polyhydroxybutyrate (PHB) production with *Paraburkholderia sacchari* IPT 101. Cellulose.

[37] Sabapathya P.C., Devaraja S., Parthiban A., Pugazhendhic A., Kathirvela P. (2019). Aegle marmelos: A novel low cost substrate for the synthesis of polyhydroxyalkanoate by *Bacillus aerophilus* RSL-7. Biocatal. Agric. Biotechnol..

[38] Dietrich K., Dumont M.J., Schwinghamer T., Orsat V., Del Rio L.F. (2018). Model Study to Assess Softwood Hemicellulose Hydrolysates as the Carbon Source for PHB Production in *Paraburkholderia sacchari* IPT 101. Biomacromolecules.

[39] Wang S., Liu Y., Guo H., Meng Y., Xiong W., Liu R., Yang C. (2024). Establishment of low-cost production platforms of polyhydroxyalkanoate bioplastics from *Halomonas cupida* J9. Biotechnol. Bioeng..

[40] Lee S.M., Cho D.H., Jung H.J., Kim B., Kim S.H., Bhatia S.K., Gurav R., Jeon J.M., Yoon J.J., Kim W. (2022). Finding of novel polyhydroxybutyrate producer *Loktanella* sp. SM43 capable of balanced utilization of glucose and xylose from lignocellulosic biomass. Int. J. Biol. Macromol..

[41] Kucera D., Pernicová I., Kovalcik A., Koller M., Mullerova L., Sedlacek P., Mravec F., Nebesarova J., Kalina M., Marova I. (2018). Characterization of the promising poly(3-hydroxybutyrate) producing halophilic bacterium *Halomonas halophila*. Bioresour. Technol..

[42] Kopf S., Root A., Heinmaa I., de Lima J.A., Åkesson D., Skrifvars M. (2024). Production and characterization of melt-spun poly(3-hydroxybutyrate)/poly(3-hydroxybutyrate-*co*-4-hydroxybutyrate) blend monofilaments. ACS Omega.

[43] Bowers T., Vaidya A., Smith D.A., Lloyd-Jones G. (2013). Softwood hydrolysate as a carbon source for polyhydroxyalkanoate production. J. Chem. Technol. Biotechnol..

[44] Jung H.J., Kim S.H., Shin N., Oh S.-J., Hwang J.H., Kim H.J., Kim Y.-H., Bhatia S.K., Jeon J.-M., Yoon J.-J. (2023). Polyhydroxybutyrate (PHB) production from sugar cane molasses and tap water without sterilization using novel strain, *Priestia* sp. YH4. Int. J. Biol. Macromol..

[45] Owen A.J., Lin J., Connell D., Beadle M., Noble M. (1992). Crystallization and melting behaviour of poly(3-hydroxybutyrate) homopolymer and its copolymer. Polymer.

[46] Gunaratne L.M.W.K., Shanks R.A. (2005). Multiple melting behaviour of poly(3-hydroxybutyrate-co-hydroxyvalerate) using step-scan DSC. Eur. Polym. J..

[47] Zinn M., Witholt B., Egli T. (2001). Occurrence, synthesis and medical application of bacterial polyhydroxyalkanoate. Adv. Drug Deliv. Rev..

[48] Whitfield R., Truong N.P., Anastasaki A. (2021). Precise control of both dispersity and molecular weight distribution shape by polymer blending. Angew. Chem. Int. Ed..

[49] Chen G.-Q., Wu Q. (2005). The application of polyhydroxyalkanoates as tissue engineering materials. Biomaterials.

