# Supplementary material for: Valorization of Dried Okara Hydrolysate for Polyhydroxybutyrate Production by Newly Isolated Burkholderia sp. EP10
Source: Bioengineering (Basel). 2026 Mar 9;13(3):313. doi: 10.3390/bioengineering13030313 (PMC13023746; doi:10.3390/bioengineering13030313)
Supplement: Supplementary file 1 [file bioengineering-13-00313-s001.zip › bioengineering-4165463-supplementary.pdf]

Supplementary Methods

Isolation of PHB producing bacterial strain

Approximately 10 g of soil was collected from a depth greater than 10 cm and suspended in 40 mL of distilled water (DW). Following vortexing, the suspension was left to settle for 15 min, after which 10 mL of the supernatant was transferred into 100 mL of MSM and incubated at 200 rpm for 3 days. This enrichment process was repeated three times, resulting in a cumulative incubation period of 9 days. Each liter of MSM was supplemented with 20 g reducing sugars derived from DOKH, obtained by hydrolyzing 13% (w/v) dried okara (DOK) with 2% H_2SO_4 at 121℃ for 30 min. The medium also included 9.0 g $Na_2HPO_4{\cdot}12H_2O$, 1.5 g KH_2PO_4, 0.5 g NH_4Cl, 0.2 g $MgSO_4{\cdot}7H_2O$, and 1 mL of trace element solution (TES). The TES was composed of 9.70 g $FeCl_3$, 10.33 g $CaCl_2{\cdot}2H_2O$, 0.22 g $CoCl_2{\cdot}6H_2O$, 0.16 g $CuSO_4{\cdot}5H_2O$, 0.12 g $NiCl_2{\cdot}6H_2O$, and 0.11 g $CrCl_2{\cdot}6H_2O$ per liter of 0.1 N HCl. Isolated bacterial strains were cultivated in MSM broth at 30°C for 24 h, mixed with an equal volume of 50% glycerol, and subsequently stored at –80°C.

Medium optimization for cell growth and PHB production

The composition of MS-E* per liter was: 7.3 g $Na_2HPO_4{\cdot}12H_2O$, 2.3 g KH_2PO_4, 0.5 g NH_4Cl, 0.05 g $MgSO_4{\cdot}7H_2O$, 0.3 g $NaHCO_3$, 0.1 g $CaCl_2{\cdot}2H_2O$, and 1 mL of trace element solution (TES). The TES was produced by mixing 0.6 g H_3BO_3, 0.58 g $ZnSO_4{\cdot}7H_2O$, 3.96 g $MnCl_2{\cdot}4H_2O$, 5.56 g $FeSO_4{\cdot}2H_2O$, 5.62 g $CoSO_4{\cdot}2H_2O$, 0.34 g $CuCl_2{\cdot}2H_2O$, 0.04 g $NiCl_2{\cdot}6H_2O$, and 0.06 g $Na_2MoO_4{\cdot}2H_2O$ in 1 L of 0.1 N HCl. The MS-H16 medium per liter consisted of 11.1 g $Na_2HPO_4{\cdot}12H_2O$, 1.0 g KH_2PO_4, 3.0 g NH_4Cl, 0.2 g $MgSO_4{\cdot}7H_2O$, and 1 mL of TES, which contained 9.7 g $FeCl_3$, 10.35 g $CaCl_2{\cdot}2H_2O$, 0.248 g $CoCl_2{\cdot}6H_2O$, 0.156 g $CuSO_4{\cdot}5H_2O$, 0.216 g $NiCl_2{\cdot}6H_2O$, and 0.139 g $CrCl_2{\cdot}6H_2O$ per liter of 0.1 N HCl. YPM was formulated with 10.0 g yeast extract, 10.0 g meat extract, 5.0 g polypeptone, and 20.0 g NaCl per liter. For inoculation, the EP10 strain was grown in LB medium, and 10% (v/v) of culture at an OD_{660} between 0.9 and 1.0 was used for inoculation of test media. Cultivation was carried out at 30°C with orbital shaking at 200 rpm for 72 h, after which cell growth and PHB accumulation were determined.

Analytical Methods

Analysis of monosaccharide composition by HPLC was conducted using a Waters Alliance 2690 system (Waters Corp., Milford, MA, USA) equipped with a Sugar-Pak I column (6.5 mm×300 mm, 10 μm) and a refractive index detector. The column temperature was maintained at 90°C, and 20 μL of the sample was injected. The mobile phase used was 0.01 M Ca-EDTA at a flow rate of 0.5 mL/min. Calibration was performed using D-glucose, D-mannose, D-xylose, and D-arabinose standards (Sigma-Aldrich, USA). Potential inhibitors, specifically 5-hydroxymethylfurfural (5-HMF) and furfural (TCI Co., Ltd., Tokyo, Japan), were quantified using a Shim-pack VP-ODS column (5 μm, 4.6 mm × 250 mm, Shimadzu Corp., Kyoto, Japan) and a mobile phase consisting of water/acetonitrile (8:2, v/v). The column was maintained at 40 °C, with a flow rate of 1.0 mL/min and a 10 μL injection volume.

Elemental composition (Na, P, Si, B, Ca, Fe, K, Mg, Mn, Zn) was determined using inductively coupled plasma optical emission spectrometry (ICP-OES) (Agilent 5110, Agilent Technologies, Santa Clara, CA, USA) with multi-element ICP standards (Ricca Chemical Co., Arlington, TX, USA). Samples were filtered, diluted appropriately, and analyzed alongside certified reference materials to ensure analytical accuracy.

For intracellular PHB quantification, 20 mg of lyophilized cells were subjected to methanolysis in a PYREX screw cap tube containing 1.0 mL chloroform, 0.85 mL methanol, 0.15 mL H_2SO_4, and 4 mg benzoic acid (internal standard) at 100°C for 3 h. Subsequent to addition of water and phase separation, the resulting chloroform layer was analyzed by gas chromatography (GC) (Shimadzu GC-2010 Plus, Kyoto, Japan) equipped with an HP-1 column and a flame ionization detector (FID). The GC oven was initially held at 80°C for 4 min, then increased to 230°C at a rate of 10°C/min. Monomer composition was further confirmed using GC-mass spectrometry (GC–MS) (Agilent 5977A, DB-5MS column).

Fourier-transform infrared (FT-IR) spectra of purified PHB were acquired using a PerkinElmer Paragon 1000 FT-IR spectrometer (PerkinElmer, Waltham, MA, USA) outfitted with an attenuated total reflectance (ATR) accessory. Finely ground PHB samples were applied directly onto the diamond ATR crystal without additional treatment. Spectra were collected over a range of 4000–400 cm^{-1}, averaging 32 scans per sample at a resolution of 4 cm^{-1}. Immediately before each analysis, background spectra were obtained and automatically subtracted. The collected spectra underwent baseline correction and normalization before interpretation.

TG/DTA analysis was conducted using a TG-DTA 8122 instrument (Rigaku Corp., Tokyo, Japan) under a nitrogen atmosphere, with the temperature increased from 20 to 900 °C at a rate of 10 °C/min to assess the thermal stability of the polymer.

For SEC analysis, approximately 5 mg of purified PHA was dissolved in 1 mL of tetrahydrofuran, filtered through a 0.45 µm PTFE syringe filter, and 50 µL of this solution was injected onto Waters Styragel HR3, HR4, and HR5E columns maintained at 35 °C. The system was comprised of a Waters Alliance e2695 SEC unit (Waters Corp.) coupled with a refractive index detector. Calibration was performed using polystyrene standards with molecular weights between 1,060 and 3,580,000 Da. Chloroform was used as the eluent at a flow rate of 1 mL/min.

Supplementary Table

Table S1. Elemental composition of DOKH obtained under optimal hydrolysis conditions (3% H_2SO_4, 121℃, 60 min). Values represent the reducing sugar fraction measured following hydrolysis.

Parameter	Concentration (g/L)
Minerals	1.060
K	0.453
Ca	0.389
Mg	0.131
Si	0.063
B	0.008
Fe	0.007
P	0.009

Supplementary Figures

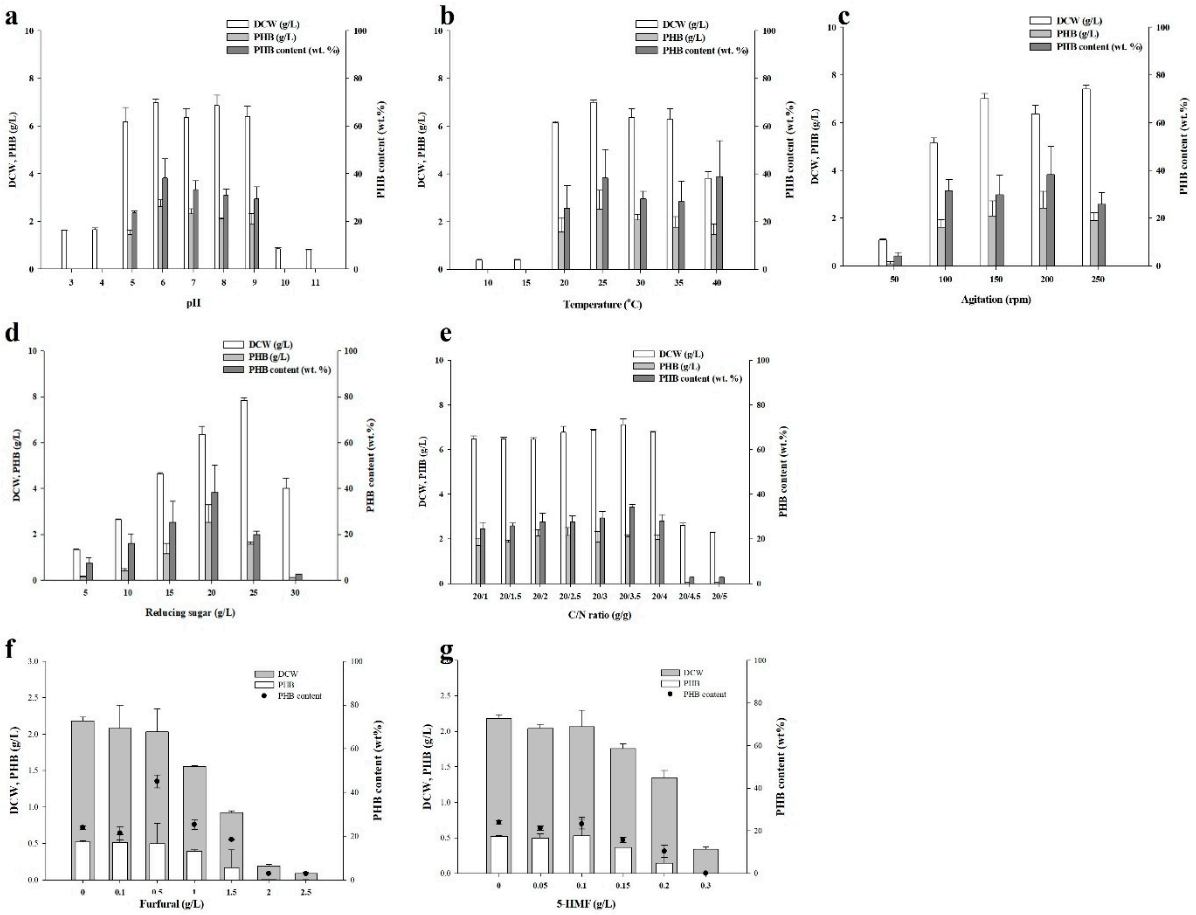

Figure S1. Effects of culture conditions and lignocellulosic inhibitors on growth and PHB biosynthesis of *Burkholderia* sp. EP10. Cultivations were conducted in DOKHM under different (a) pH values, (b) temperatures, (c) agitation speeds, (d) reducing sugar concentrations, and (e) C/N ratios. For inhibitor tolerance analysis, cells were cultivated in MSM containing xylose (20 g/L) for 72 h with (f) furfural or (g) 5-HMF. Data represent cell growth, PHB content, and PHB concentration.

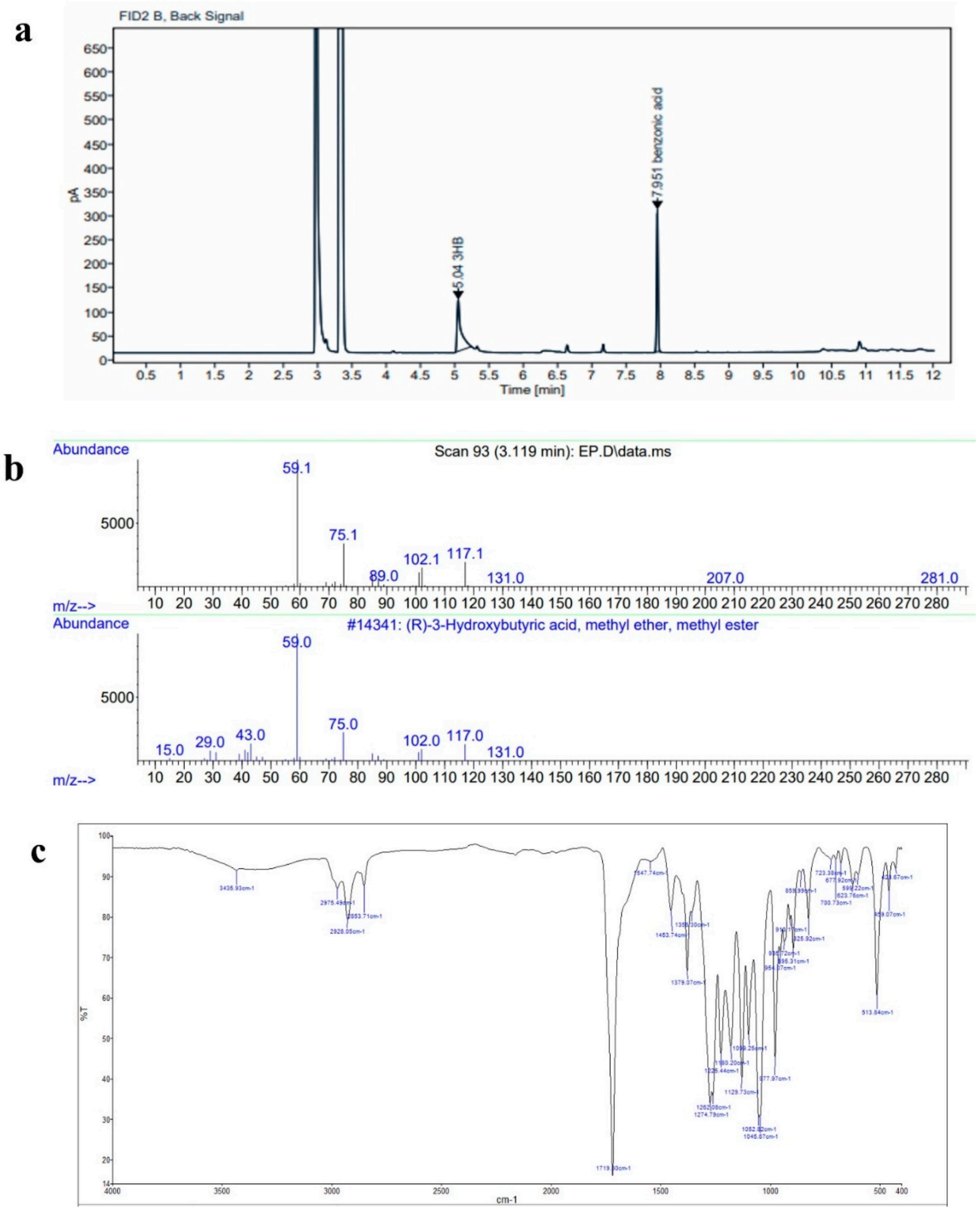

Figure S2. Structural characterization of PHB isolated from *Burkholderia* sp. EP10 grown in DOKHM medium with a C/N ratio of 5.7, initiated at pH 6 without subsequent pH adjustment. Following fermentation and cell collection, PHB was extracted and subjected to (a) GC and (b) GC–MS analysis, employing benzoic acid as the internal standard for determining monomer composition, and (c) FT-IR spectroscopy to verify functional groups and structural features.

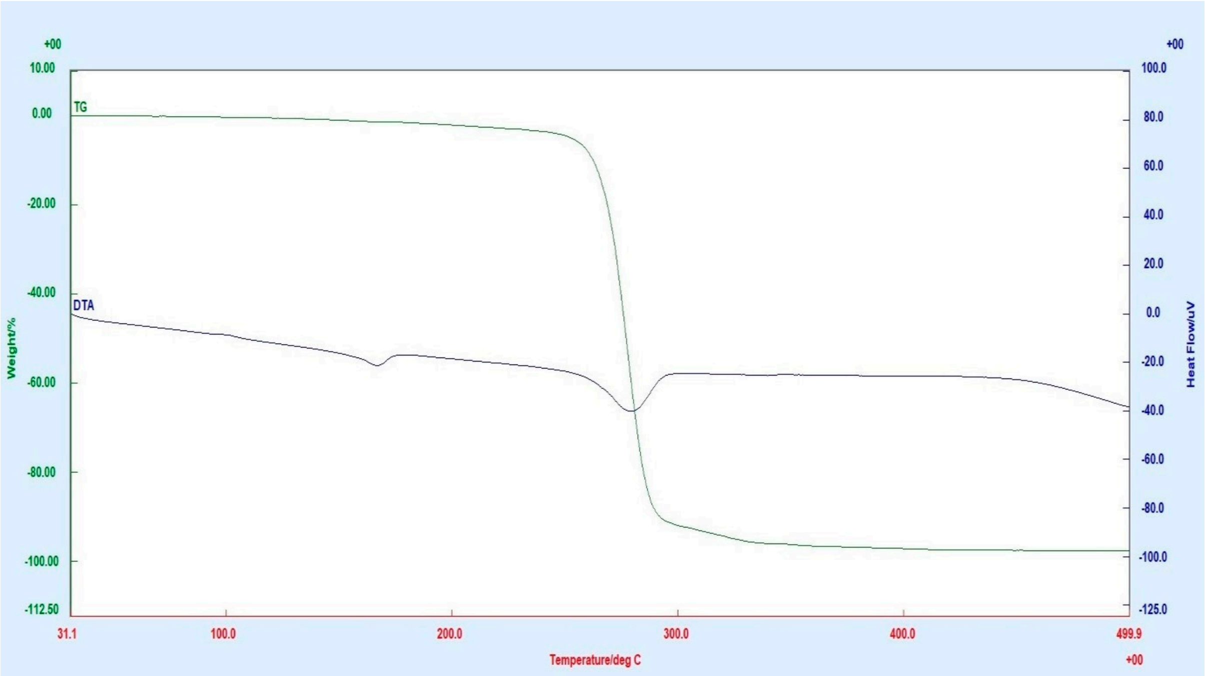

Figure S3. Thermal stability assessment of PHB isolated from *Burkholderia* sp. EP10 grown in DOKHM medium at a C/N ratio of 5.7, initiated at pH 6 without further pH regulation. After fermentation and PHB isolation, TG–DTA analysis was conducted to assess the thermal degradation profile.

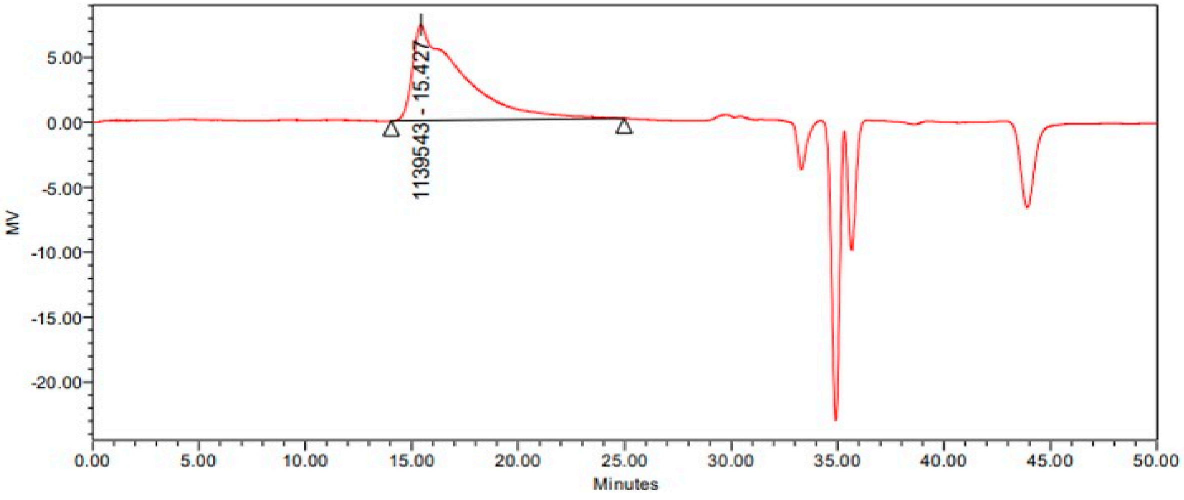

Figure S4. Molecular weight profile of PHB isolated from *Burkholderia* sp. EP10 grown in DOKHM medium at a C/N ratio of 5.7, initiated at pH 6 without additional pH regulation. PHB samples were examined by SEC to assess M_w, M_n, and PDI.
